# Analysis of bond strength between a nylon reinforcement structure and dental resins

**DOI:** 10.4317/jced.57654

**Published:** 2021-05-01

**Authors:** Leonardo-Jiro-Nomura Nakano, Guilherme-da Rocha-Scalzer Lopes, Aline-Silva Firmino, Jefferson-David-Melo de Matos, Rubens-Nisie Tango, Tarcisio-José-de Arruda Paes-Junior

**Affiliations:** 1 Department of Prosthodontics and Dental Materials, São Paulo State University (Unesp) – Institute of Science and Technology, São José dos Campos - SP, Brazil; 2 Department of Dentistry, Universidade São Francisco UFS, Bragança Paulista – SP, Brazil

## Abstract

**Background:**

Nylon is a polymer that its use to reinforce dental resins has shown positive results such as increased flexural strength. The aim of this study was to evaluate the bond strength between dental resins and a nylon reinforcement.

**Material and Methods:**

Forty cylindrical nylon blocks with 13 x 23 mm with 0.5% by volume of silica and 40 without were made. Half of the samples of each nylon composition were sandblasted with aluminum oxide (50μm) for 3 s (2.8 bar pressure, distance: 20 mm, incidence angle: 90o). On the nylon blocks, cylinders of chemically activated acrylic resin and indirect composite resin were made, with a bonding area of 6,28 mm2. Eight different groups were obtained according to the material used and the surface treatment (n = 10): Acrylic Resin + Nylon; GAS: Acrylic Resin + Nylon with Silica; GAT: Acrylic Resin + Nylon (Al2O3); GAST: Acrylic Resin + Nylon with Silica (Al2O3); GC: Composite Resin + Nylon; GCS: Composite Resin + Nylon with Silica; GCT: Composite Resin + Nylon (Al2O3); GCST: Composite Resin + Nylon with Silica (Al2O3). The shear test was carried out. The Student’s and the Kruskal-Wallis test was adopted.

**Results:**

There was no statistically difference in the bond strength for nylon with silica for the acrylic resin group. For the composite groups, nylon with silica did not present a statistically difference without surface treatment (*p* = 0.10) and with surface treatment the bond strength decreased (*p* = 0.000). The GCT showed a higher bond strength (0.89 MPa). The surface treatment improved the bond strength for the both groups.

**Conclusions:**

The presence of silica in the nylon composition did not influence the bond strength between materials evaluated. However, the surface treatment with aluminum oxide proved to be favorable for this adhesion.

** Key words:**Nylons - Resins, Synthetic - Structures Strengthening - Dental Research.

## Introduction

Since the introduction of acrylic resin, or polymethylmethacrylate (PMMA), in dental practice, there is a continuous search to change processing techniques and their composition to improve their physical and mechanical properties. Even so, this material is widely used in dentistry for its qualities such as biocompatibility, easy handling, reliability, stability in the oral environment, color stability, favorable aesthetics and low cost (1,2). However, despite these advantages, this material has some characteristics that can be improved for a better clinical performance, including flexural strength, maximum fracture load resistance and hardness (3-5).

The lowest flexural strength of acrylic resin denture bases for interocclusal records is considered the main factor of clinical failures (6,7). Several methods have been developed to increase the strength of the material such as plasticization, copolymerization with rubber, use of metal reinforcement and the use of fibers (8-10). In the last few years, there has been a considerable increase in the use of fiber-reinforced composites, especially glass, aramid, carbon, polyester, polyethylene and nylon fibers (11-23,24).

The use of the fibers has as main objective to overcome the mechanical limitations of the polymers. The fiber reinforcement behavior depends on the percentage of material added, the length and orientation of the fibers, and adhesion between fibers and resin. The fiber arrangement can be used in different directions, for example, unidirectional or bidirectional, changing the strength of the material (24,25).

Nylon is a thermoplastic polymer of the polyamide class, and it is produced by the condensation reaction between a diamine and a dibasic acid. It’s useful in the dental resins is due to their durability and strength properties (24). It can also be noted that the use of this polymer to reinforce composite resins has shown positive results such as increased flexural strength (26-28).

The nylon surface treatment is a factor that can contribute to the bond between nylon and dental resins, improving its mechanical behavior. In this sense, the presence of silica causes the mesh to chemically bond to polymeric materials (29). However, there is still no study on the contribution of silica to the nylon structure with regard to adhesion. Therefore, the aim of this study was to analyze the bond strength between dental resins and silica-nylon reinforcement.

## Material and Methods

Forty cylindrical nylon blocks with 13 x 23 mm (Natmar Moldes e Plásticos Ltda company) were made. In addition, 40 samples were developed with the same material incorporating 0.5% by volume of silica (ICT / UNESP, São José dos Campos, Patent nº: BR1020120281198) (21,30).

Half of the samples of each nylon composition were sandblasted with aluminum oxide (50μm) for 3 s (2.8 bar pressure, distance: 20 mm, incidence angle: 90o).

**Figure 1:**
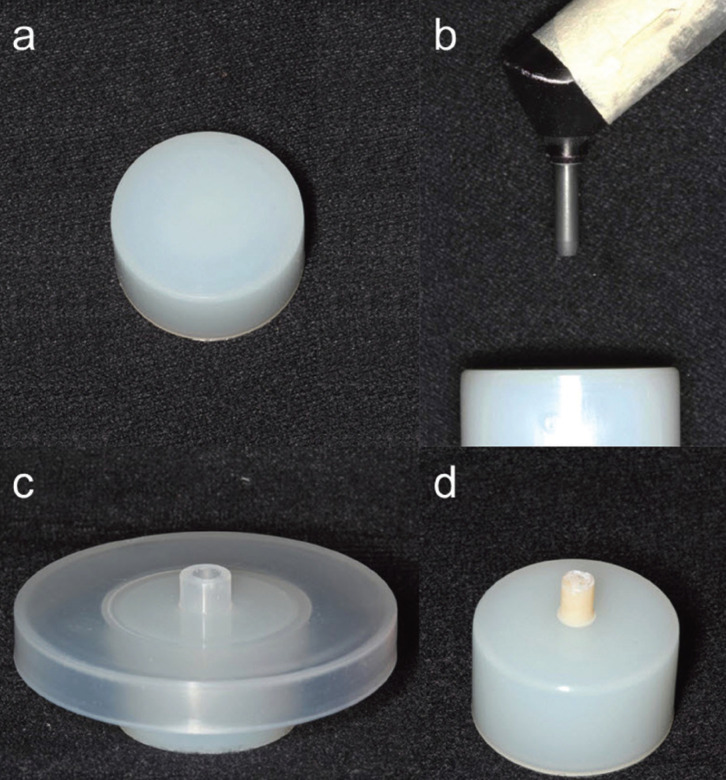
Specimen preparation. a) nylon base; b) nylon base positioned for surface treatment; c) teflon matrix; d) finished sample (nylon base + composite or acrylic resin).

On the nylon blocks, cylinders of chemically activated acrylic resin (Vipi-Flash, VIPI Produtos Odontológicas) and indirect composite resin NanolabZ (WILCOS do Brasil Ind. E Com. Ltda) were made using a teflon matrix, with a bonding area of 6,28 mm2. The composite resin was deposited in increments and light-cured for 2 min under vacuum in the Visio Beta Vario oven (3M ESPE, Seefeld, Germany), following the manufacturer’s recommendations (Fig. 1).

Eight different groups were obtained according to the material used and the surface treatment (n = 10) (Table 1): GA: Acrylic Resin + Nylon; GAS: Acrylic Resin + Nylon with Silica; GAT: Acrylic Resin + Nylon (Al2O3); GAST: Acrylic Resin + Nylon with Silica (Al2O3); GC: Composite Resin + Nylon; GCS: Composite Resin + Nylon with Silica; GCT: Composite Resin + Nylon (Al2O3); GCST: Composite Resin + Nylon with Silica (Al2O3).

**Table 1:**
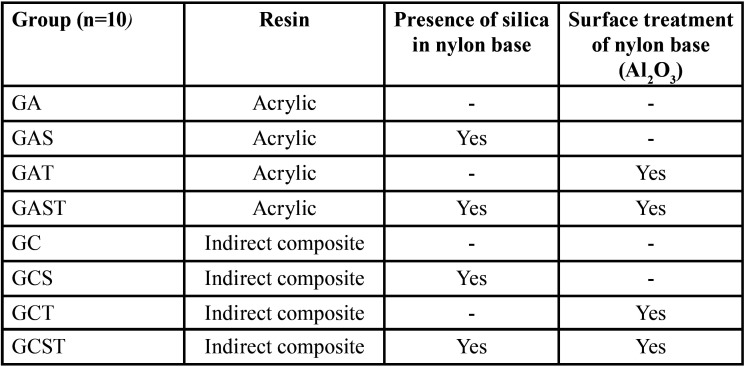
Distribution of groups according to the type of resin, presence of silica in nylon base and surface treatment.

**Figure 2:**
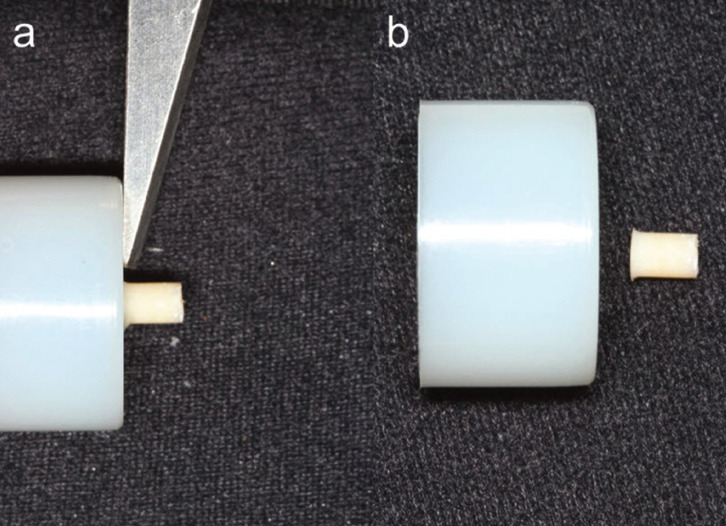
Shear bond test. a) sample positioned for the shear bond test; b) sample after performing the test.

After 24 hours of polymerization, the shear test was carried out in a universal testing machine (EMIC DL 1000, São José dos *Pi*nhais, Brazil). For the test, the samples were fixed to a metallic device, where a knife chisel directed perpendicularly carried out the loading. The movement was automatically stopped when the specimens debonded (Fig. 2).

-Failure and statistical analysis

The fractured specimens were examined by stereomicroscope (Stereo Discovery V20, Zeiss, Göttingen, Germany), with a 30× magnification and the failures were classified as an adhesive (resin cement totally present in the composite resin), predominantly adhesive (60% or higher amount of cement in the composite resin) or cohesive. Only the predominantly adhesive and adhesive fractures were considered for statistical analysis. The bond strength (MPa) was calculated by dividing the failure load (N) by the adhesive area (mm2).

The present study evaluated two groups for each factor: material (PMMA x composite), silica (added or not) and surface treatment (blasted or not). A normality analysis was applied (assumption of several parametric tests) for the bond strength data with Shapiro-Wilk’s test and for a normal distribution data the Student’s t-test was adopted and for a non-normal distribution data the Kruskal-Wallis test was adopted.

Statistical tests were performed in R-project 3.2.0 statistical software (R Core Team, Vienna, Austria). The level of significance established for the tests was 5%, which established a 95% confidence interval for the presented results, and the power of a statistical test was 80%.

## Results

**Figure 3:**
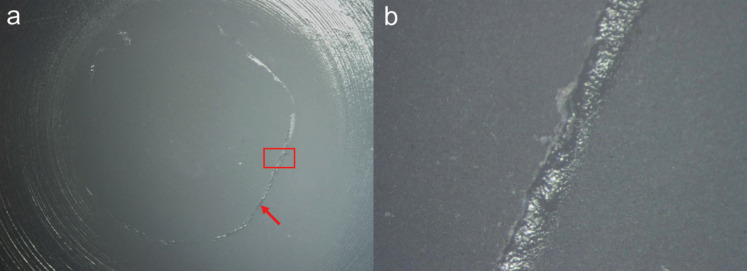
Stereomicroscopic image of the representative predominantly adhesive failure of the GAT specimen under 7.5x magnification (a), and the area delimited in red under 67x magnification (b); acrylic resin (red arrow).

**Figure 4:**
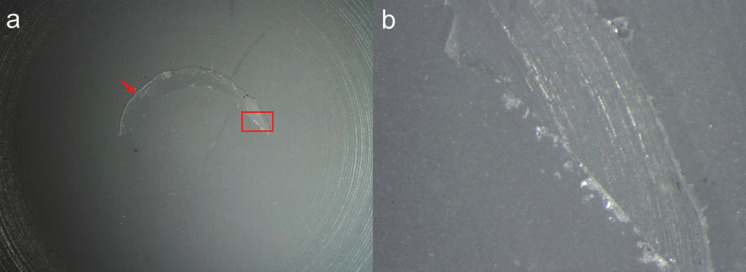
Stereomicroscopic image of the representative predominantly adhesive failure of the GCT specimen under 7.5x magnification (a), and the area delimited in red under 67x magnification (b); composite resin (red arrow).

Regarding the failure mode distribution, a predominantly adhesive and adhesive were observed for all specimens, as shown in Figures 3 and 4.

There was no statistically difference in the bond strength data for nylon with silica for the acrylic resin group without (*p* = 0.14) and with surface treatment (*p* = 0.83). For the composite groups, nylon with silica did not present a statistically difference without surface treatment (*p* = 0.10) and with surface treatment the bond strength decreased (*p* = 0.000).

The composite showed a higher bond strength (0.89 MPa) with a statistically difference of acrylic resin (0.24 MPa) (*p* = 0.000). The surface treatment improved the bond strength for the both groups. For the acrylic resin group without (*p* = 0.005) and with silica (*p* = 0.000); and for the composite group without (*p* = 0.000) and with silica (*p* = 0.001). Thus, the statistically tests presented the difference between the groups. In Table 2, it is possible to verify the results by correlating the factors (material, presence of silica and surface treatment) and the bond strength means of homogeneous groups

**Table 2:**
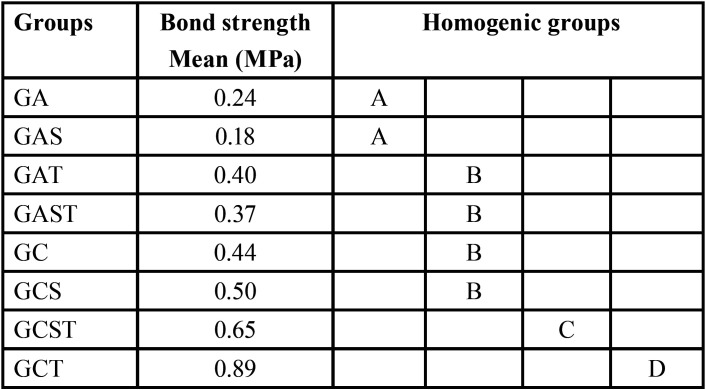
Bond strength means of homogeneous groups.

## Discussion

In the present study, the composite resin group that does not have silica and has undergone surface treatment obtained greater bond strength values, and the groups in which they were subjected to surface treatment, regardless of whether they have silica or not, showed the best results. This result corroborates with what was obtained in the acrylic resin groups and reinforces the effectiveness of the surface treatment in relation to the increase of the bond strength between the studied materials.

There are no studies that performed the nylon surface treatment, therefore, the sandblasting methodology was adopted (16). The methodology of this study used a shear test to evaluate the bond strength, already used in several studies (11,14,15). Only samples that showed real adhesive values (31), being adhesive or predominantly adhesive, were included in the statistical computation.

In previous studies, the added of silica in the nylon mesh presented an increase in fracture load, flexural strength (21,23), and bond strength (29). According the adhesion properties, the presence of silica can improve the chemical bonding with the polymeric radical of the resins. Despite this, it is assumed that one of the reasons for this difference in the results obtained was the influence of the shape and architecture of the specimens, in which, depending on the structure used, there may be a difference in the amount of silica in contact with polymers, such as in previous studies where a nylon grid system was used as reinforcement material and obtained positive results with the presence of silica in nylon (21,23).

The surface treatment was evaluated, where aluminum oxide sandblasting was carried out to verify its influence on nylon adhesion with dental polymers. Corroborating the study by other, the surface treatment increases the roughness and the surface area, allowing greater micromechanical adhesion through the imbrication between the polymer and the nylon surface (32).

The results obtained (Table 2) revealed that the groups where the nylon surface treatments were performed achieved the highest bond strength values. It is assumed that the sandblasting may have activated the silane present in the nylon structure, which positively influenced the result, in addition to providing an increase in the surface area, which improve the adhesion between the materials (21,23). Silane is essential for the charge particles of the composite resin to remain adhered to the resinous matrix, and this allows the polymeric matrix to transfer tensions to the charge particles, which are more rigid (1).

In other study (21), the fracture strength of temporary resins was evaluated, using a nylon mesh with and without silica as reinforcement, and the group in which the nylon contained silica showed the higher strength values. Within the limitations of the study, the presence or not of silica did not improve the bond strength, but the indication of maintaining silica to improve this adhesion is not discarded. Other studies evaluating the architecture of the nylon mesh and the silica distribution are necessary to investigate possible influences on bond strength.

In this study, although the main proposal was to verify the influence of silica on the bond strength, it was found that the surface treatment was a major factor than others. Thus, it can be suggested the surface treatment with aluminum oxide in nylon reinforcement systems containing silica, regardless of the dental polymer, to improve the bond strength.

## Conclusions

It can be concluded that:

The presence of silica in the nylon composition did not improve the bond strength between the nylon and the evaluated resins. However, the surface treatment with aluminum oxide proved to be favorable for this adhesion.
